# THE CORRELATION BETWEEN INTRINSIC CAPACITY AND FALLS AMONG COMMUNITY-DWELLING OLDER ADULTS AND ITS APPLICATION

**DOI:** 10.1093/geroni/igae098.1045

**Published:** 2024-12-31

**Authors:** Chen Li, Xiaodong Chen, Lingxiao He

**Affiliations:** Center for Health Economics, Policy and Aging Research, Xiamen city, Fujian, China; Center for Aging and Health Research, School of Public Health, Xiamen University, Xiamen, China, Ximen, Fujian, China; Center for Health Economics, Policy and Aging Research, Xiamen city, Fujian, China

## Abstract

**Objective:**

To explore the link between intrinsic capacity and fall risk among Xiamen’s older adults and to assess the diagnostic value of intrinsic capacity composite scores for falls.

**Methods:**

A survey covering intrinsic capacity and fall history was conducted on 612 Community-Dwelling older adults. Logistic regression and ROC curve analyses determined the intrinsic capacity composite score’s cutoff for fall risk.

**Results:**

Higher scores indicated lower fall risk （OR=0.45,95%CI: 0.30-0.67）. The optimal cutoff value was identified as 0.367, with the maximum AUC of 0.689. Adding chronic diseases as a control slightly reduced the AUC to 0.682 but did not change the cutoff.

**Conclusion:**

Intrinsic capacity is a protective factor against falls. A score of 0.367 effectively diagnostic fall risk, aiding in older adults` fall prevention strategies.

